# Increased Expression of *TGFβR2* Is Associated with the Clinical Outcome of Non-Small Cell Lung Cancer Patients Treated with Chemotherapy

**DOI:** 10.1371/journal.pone.0134682

**Published:** 2015-08-07

**Authors:** Yang Han, Chengyou Jia, Xianling Cong, Fei Yu, Haidong Cai, Suyun Fang, Li Cai, Huiqiong Yang, Yu Sun, Dan Li, Jin Liu, Ruting Xie, Xueyu Yuan, Xiaoming Zhong, Ming Li, Qing Wei, Zhongwei Lv, Da Fu, Yushui Ma

**Affiliations:** 1 Department of Nuclear Medicine, Shanghai 10th People’s Hospital, Tongji University School of Medicine, Shanghai, China; 2 Tissue Bank, China-Japan Union Hospital, Jilin University, Changchun, China; 3 Department of pathology, Shanghai 10th People’s Hospital, Tongji University School of Medicine, Shanghai, China; 4 Department of Radiology, Jiangxi Provincial Tumor Hospital, Nanchang, China; 5 Department of Respiratory Medicine, Shanghai 10th People’s Hospital, Tongji University School of Medicine, Shanghai, China; University of North Carolina School of Medicine, UNITED STATES

## Abstract

To investigate the prognostic significance of *TGFβR2* expression and chemotherapy in Chinese non-small cell lung cancer (NSCLC) patients, *TGFβR2* expression NSCLC was analyzed *in silico* using the Oncomine database, and subsequently analyzed with quantitative RT-PCR in 308 NSCLC biopsies, 42 of which were paired with adjacent non-neoplastic tissues. Our results show that *TGFβR2* expression was also increased in NSCLC biopsies relative to normal tissue samples and correlated with poor prognosis. *TGFβR2* expression was also significantly correlated with other clinical parameters such as tumor differentiation, invasion of lung membrane, and chemotherapy. Moreover, overall survival (OS) and disease free survival (DFS) was increased in patients with low *TGFβR2* expressing NSCLC and who had undergone chemotherapy. Thus, high expression of *TGFβR2* is a significant risk factor for decreased OS and DFS in NSCLC patients. Thus, *TGFβR2* is a potential prognostic tumor biomarker for chemotherapy.

## Introduction

Lung carcinoma is the leading cause of cancer-related mortality worldwide, accounting for 1.37 million deaths annually [1–3]. Non-small cell lung cancers (NSCLC) are the most common type of primary lung cancer, accounting for almost 80% of lung carcinoma [4]. Although significant advances have taken place in our understanding of the disease process during the past few decades [5,6], the main treatment strategy is still surgical resection, chemotherapy, and radiation therapy [7–9]. However, even in the case of complete resection, curative effect is not satisfactory and NSCLC patients still face the risk of recurrence and metastasis [10,11].

The hope for complete molecular analysis of human cancers is ultimately to improve the management of patients. Advances in genomics and proteomics have generated many candidate markers with potential clinical value [12]. Therefore, whether biomarkers exist that would function as predictive factors for lung carcinoma or be used in the decision making process for clinical management of patients is currently under investigation as options for treatment advance. Furthermore, understanding the molecular characteristics of lung cancers would aid in targeted therapy development.

Components of the transforming growth factor-beta (TGF-β) family are often altered in the development of various human cancers. TGF-β is a pleiotropic cytokine, which acts as a tumor suppressor or tumor promoter depending upon the cellular microenvironment [13]. TGF-beta receptor type-2 (TGFβR2) is the ligand-binding receptor for all members of the TGF-β family [14–16]. TGFβ signals are mediated by an activated complex of TGFβR1 and TGFβR2 [17]. The TGF-β ligand primarily binds to TGFβR2 at the plasma membrane, resulting in the formation of a complex between TGFβR1 and TGFβR2. TGFβR2 phosphorylates TGFβR1, and activated TGFβR1 phosphorylates downstream targets, Smad2 and Smad3. Phosphorylated Smad2 and Smad3 form a complex with Smad4, which translocates to the nucleus and regulates target gene expression [18,19]. Therefore, abnormalities in any member of the TGF-β or Smad family often profoundly disrupt the TGF-beta signaling pathway [20,21].

Whether a gene signature can predict clinical outcome of NSCLC, including prognosis and response to chemotherapy, remains unclear. Here, the Oncomine database was used to reveal differential expression specifically of TGFβR2 in NSCLC. The expression of TGFβR2 was subsequently validated by real-time PCR in NSCLC biopsies from a cohort of Chinese patients and prognostic significance was assessed.

## Materials and Methods

### Ethics statement

The study was reviewed and approved by the Ethical Committee of Jilin University (Jilin, China). Every participant provided their written informed consent to participate in this study and the ethics committees approve the consent procedure.

### Set-up of server for online survival calculation

*TGFβR2* mRNA expression was investigated in NSCLC tissue samples (n = 187) in the TCGA database through the Oncomine Platform (http://www.oncomine.org). Data were retrieved by using search terms “*TGFβR2*” and “NSCLC” and “mRNA”.

The prognostic value of the *TGFβR2* gene was assessed with the Kaplan Meier plotter, a meta-analysis tool based in silico biomarker assessment, which assesses the effect of genes on survival in cancer patients (http://www.kmplot.com/lung) [22,23]. Each median was computed and the best performing threshold was used as the final cutoff in a univariate and Cox regression analysis. Histology, grade, stage, gender, and smoking history were used in the multivariate analysis. A Kaplan-Meier survival plot and the hazard ratios with 95% confidence intervals and the log rank *P* value were calculated. Significance was set at *P*< 0.05.

### Acquisition of clinical specimens

Fresh tissue samples from NSCLC patients who underwent surgical resection between 2008 and 2012 were obtained from the tissue bank, Jilin University (Jilin, China). Samples included paired tumor and adjacent non-cancerous tissues (n = 42) as well as a large cohort of individual NSCLC biopsies (n = 266). All tumors were staged according to the 7th edition of the AJCC TNM staging system for NSCLC, and patient data collected up to April 30, 2014 were included for all patients. Clinical data recorded included patient characteristics (e.g., gender, age), tumor characteristics (e.g., diameter, lymph-node metastasis, histological subtypes, vascular invasion, and tumor differentiation), overall survival (OS) and disease-free survival (DFS), *TGFβR2* expression status, and chemotherapy.

For analysis, patients were stratified according to age, ≥ 60 or < 60 years. Tumor size was defined as the mean tumor diameter (MTD, defined as the geometric mean of four diameters on the CT scan), and tumors were grouped according to size, ≥ 5 cm and < 5 cm. The follow-up was conducted by telephone or direct correspondence. The time to tumor relapse or death was confirmed by the patient or relatives, by medical recording, or by the social security record. Overall survival (OS) was calculated in months from the date of diagnosis to the time of death, regardless of cause. Disease free survival (DFS) was defined as the period from the initial date of diagnosis to the time of tumor progression by CT scan, or to the time of death due to the disease.

### RNA Extraction

Total RNA was extracted from NSCLC and normal tissue with TRIzol reagent, according to the manufacturer’s instructions. RNA concentration was measured in a spectrophotometer, and the quality of all RNA samples was assessed by electrophoresis on 1.5% denaturing agarose gels.

### Quantitative RT-PCR

For quantitative real-time PCR (qRT-PCR), cDNA was synthesized from total RNA (10 ng), and quantitative PCR reactions were performed with the Taqman Universal PCR Kit. GAPDH was used as the internal control. The 2^-δδCT^ method was used to quantify the expression levels of *TGFβR2*.

### Statistical analysis

All statistical analyses were performed with IBM SPSS statistics for Windows, Version 19.0. The expression of *TGFβR2* was presented as the mean ± standard deviation. An independent T-test was used to examine differences between two groups, and a chi-square test was used to evaluate differences in rates between groups. Kaplan-Meier curves were used to determine overall survival of the various groups, and results were compared with a log-rank test. Univariate and multivariate survival analyses were based on a Cox regression model, and this model was used to identify which independent factors jointly had significant effects on survival. *P*< 0.05 were considered statistically significant.

## Results

### Expression of the TGFβR2 using online survival analysis platform

**Fig 1:**
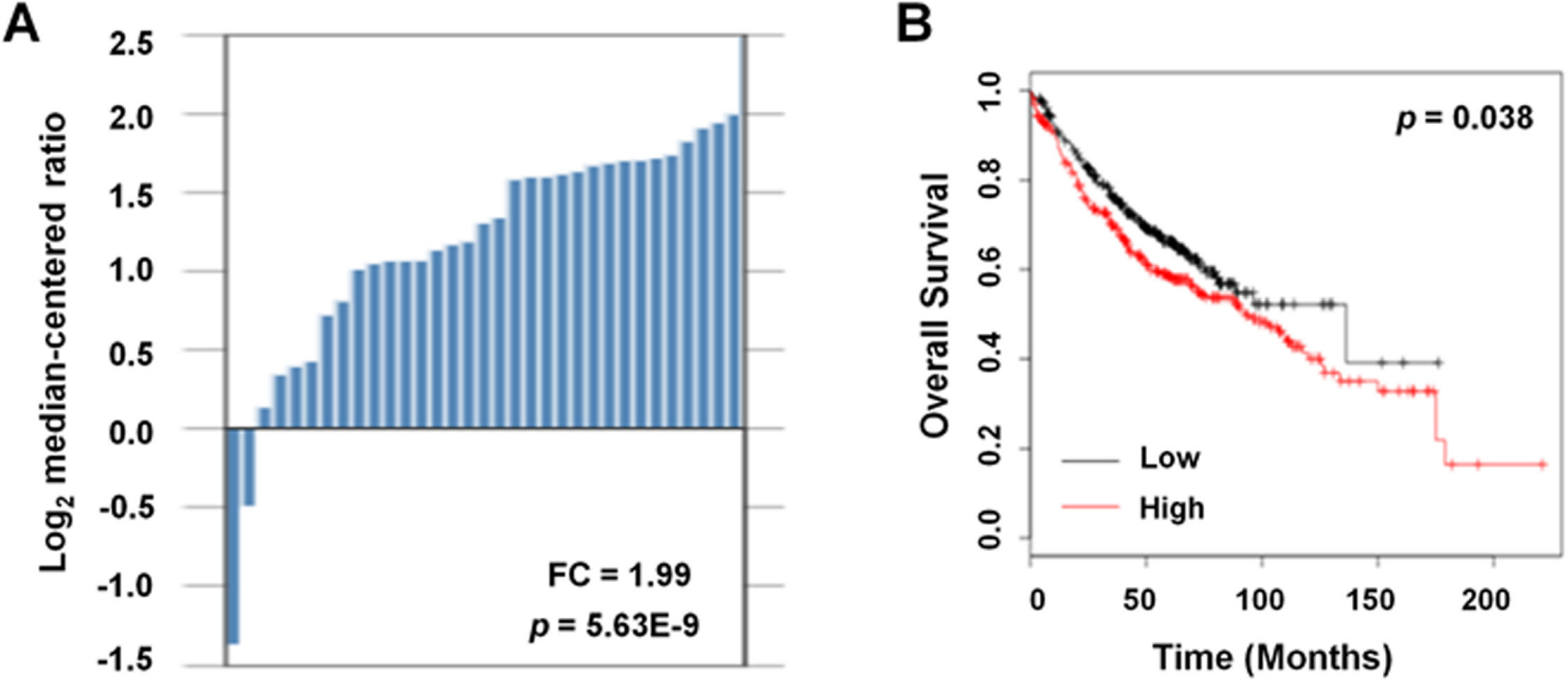
Analysis of *TGFβR2* gene expression of the in NSCLC patients with Oncomine, an online survival analysis platform. A, Expression levels of *TGFβR2* in cancer vs. normal tissues derived from the Oncomine database. B, Survival analysis performed with Kaplan-Meier plots based on *TGFβR2* expression.

*TGFβR2* mRNA expression in cancer vs. normal tissues was investigated using the Oncomine database (Fig 1A). This analysis revealed that *TGFβR2* was over-expressed in tumor tissues as compared to the corresponding normal tissue (fold change = 1.99; *P*< 0.001).

The prognostic value of the expression of *TGFβR2* was assessed with the Kaplan-Meier plotter platform. The results demonstrated that prognosis was worse for patients with lung adenocarcinoma where *TGFβR2* expression was >1.99 fold higher than in normal tissues (HR = 1.28 (95% CI, 1.01–1.63), *P* = 0.038). These results indicated that high expression of *TGFβR2* (> 1.99 fold) was associated with a poor prognosis in lung adenocarcinoma (Fig 1B).

### TGFβR2 expression in NSCLC and normal lung tissue

To validate these findings, *TGFβR2* mRNA levels were examined in tumor (n = 308) and adjacent non-cancerous tissues (n = 42) from a cohort of Chinese NSCLC patients by qRT-PCR. The results demonstrated that *TGFβR2* expression levels were significantly higher in NSCLC tumor biopsies (2.46 ± 0.42) relative to adjacent non-neoplastic tissues (1.01 ± 0.06). This difference between tumor and normal tissues was statistically significant (*P* = 0.001; Table 1).

**Table 1:** TGFβR2 expression in normal lung and NSCLC tissues.

Group	No.	TGFBR2 (Mean ± SD)	P value
Normal lung tissue	42	1.01 ± 0.06	0.001
Lung cancer tissue	308	2.46 ± 0.42	

### The relationships between TGFβR2 expression and clinical characteristics

Associations between *TGFβR2* expression and individual clinical characteristics were investigated. The results demonstrated that in these 308 cases of NSCLC, *TGFβR2* expression levels were positively correlated with tumor differentiation (*P* = 0.029) and invasion of the lung membrane (*P* = 0.045) as well as chemotherapy (*P* < 0.001; Table 2).However, there was no association between *TGFβR2* expression and patient gender, age, smoking history, lymph-node metastasis, histology, vascular invasion, TNM stage, or tumor diameter (*P* >0.05).

**Table 2:** Univariate analysis of overall survival based on patients stratified by clinical characteristics.

Factor	Varible	No.	TGFβR2 experssion	P value		Overall survival			
			(Mean ± SD)		Months (Mean)	95% CI (Mean)	P value^#^
Age							
	≥ 60	168	0.87 ± 0.15	0.139	25.03	22.61–27.44	0.772
	< 60	140	0.65 ± 0.10		28.26	25.45–31.06	
Gender							
	Male	191	0.73 ± 0.92	0.442	27.16	24.80–29.52	0.101
	Female	117	0.84 ± 0.12		25.31	22.44–28.18	
Smoking history							
	Never	208	0.78 ± 0.89	0.886	26.49	24.34–28.65	0.848
	Ever	100	0.75 ± 0.13		26.38	22.97–29.79	
Lymphnode metastasis							
	Negative	141	0.83 ± 0.81	0.396	30.48	27.42–33.54	0.026
	Positive	157	0.71 ± 0.13		22.60	20.60–24.61	
	Unknow	10					
Tumor differentiation							
	Poorly	3	2.34 ± 0.67	0.029	29.09	4.60–53.58	0.416
	Moderately	183	0.82 ± 0.11		27.68	25.28–30.08	
	Well	122	0.66 ± 0.11		24.56	21.70–27.41	
Histology							
	Adenocarcinoma	203	0.72 ± 0.83	0.32	26.70	24.39–29.01	0.07
	Squamous cell carcinoma	105	0.87 ± 0.14		25.99	23.02–28.95	
TNM stage							
	I	225	0.79 ± 0.65	0.564	28.32	26.08–30.56	0.226
	II	83	0.71 ± 0.22		21.41	18.71–24.11	
Invasion of lung membrane							
	Negative	10	1.04 ± 0.22	0.045	33.16	20.07–46.25	0.034
	Positive	221	0.67 ± 0.06		24.32	22.31–26.34	
	Unknow	76					
Vascular invasion							
	Negative	295	0.77 ± 0.75	0.869	26.36	24.51–28.21	0.11
	Positive	2	0.97 ± 0.54		15.22	23.53–53.97	
	Unknow	11					
Chemotherapy							
	Negative	57	0.19 ± 0.22	< 0.001	20.11	17.48–22.74	0.033
	Positive	70	1.46 ± 0.19		25.85	23.48–28.21	
	Unknow	181					
Diameter							
	≥ 5 cm	48	0.75 ± 0.14	0.882	19.56	16.50–22.62	< 0.001
	< 5 cm	260	0.78 ± 0.08		27.73	25.68–29.78	

# Log—rank test.

Kaplan-Meier survival curves were plotted in order to evaluate the prognostic value of these clinical/biological characteristics for OS. The median follow-up was 34.3 months (range from 14.3 to 79.3 months). The results of univariate analyses are shown in Table 2. As expected, there was a significant association between shorter OS and classical prognostic factors such as lymph-node metastasis (*P* = 0.026), invasion of lung membrane (*P* = 0.034), and tumor size (≥ 5 cm; *P* = 0.033). Furthermore, patients treated with chemotherapy displayed a significantly increased OS (*P*< 0.001). Thus, significantly decreased OS was associated with patients who had not undergone chemotherapy or had lymph node metastasis, invasion of lung membrane, or increased tumor size (≥ 5 cm).

### High expression of TGFβR2 was a prognostic marker for NSCLC patient survival

**Fig 2:**
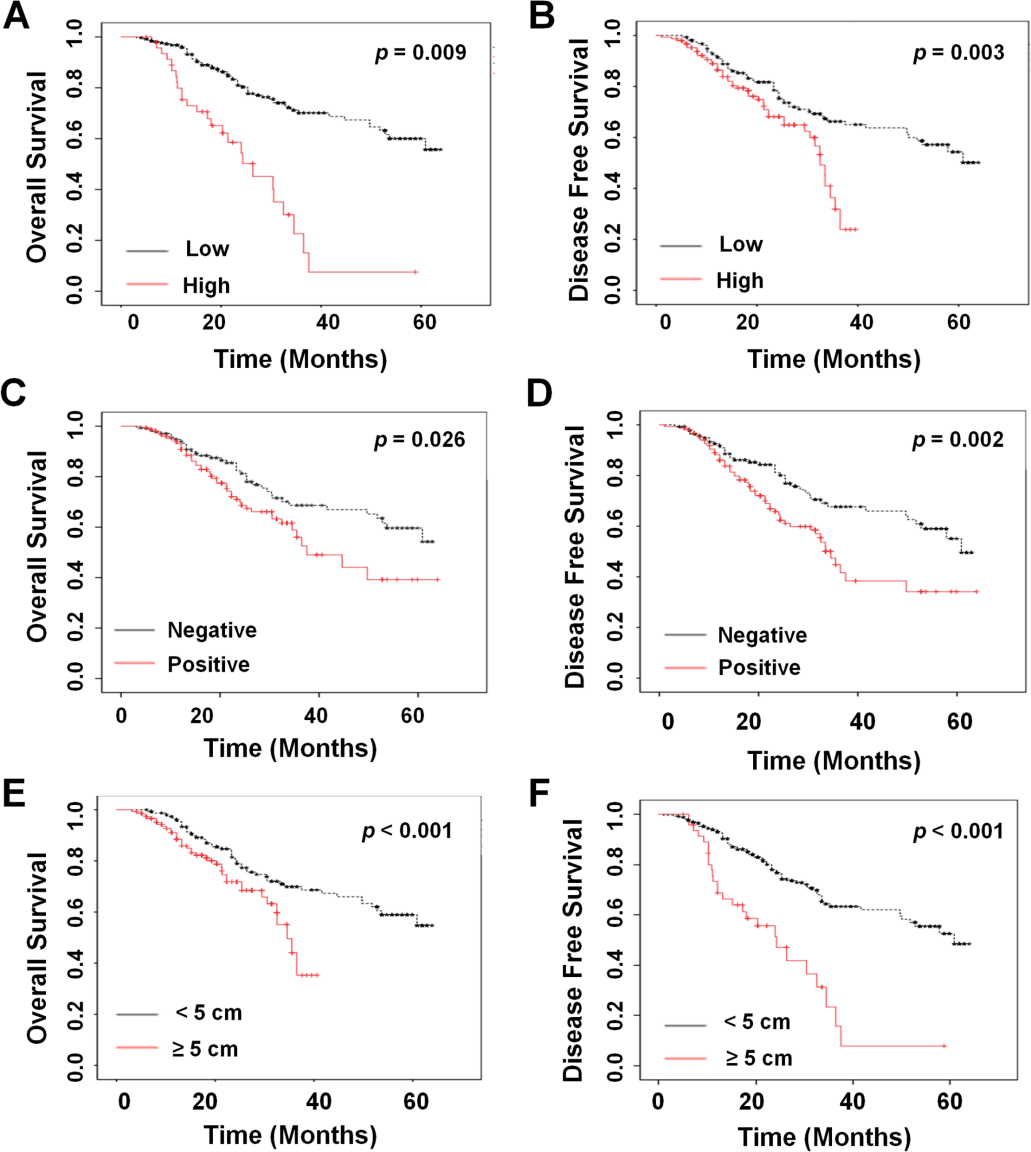
Univariate survival analysis of different clinical parameters in NSCLC. Univariate survival analysis of OS and DFS in lung carcinoma as determined by Kaplan-Meier plots estimates based on *TGFβR2* expression in (A) and (B); lymph-node metastasis in (C) and (D); and lung carcinoma tumor size in (E) and (F), respectively.

To determine whether other clinical factors might affect the prognosis of NSCLC, univariate survival analysis, stratified by each of the clinical factors (including gender, age, vascular invasion, tumor size, invasion of lung membrane, lymph-node metastasis, TNM stage, tumor differentiation, smoking story, and *TGFβR2* expression), was performed with Kaplan-Meier estimates. The results of Kaplan–Meier survival analysis demonstrated that prognosis was worse in patients with high expression of *TGFβR2*. Expression of *TGFβR2* was significantly associated with decreased OS (*P* = 0.009; Fig 2A) and DFS (*P* = 0.003; Fig 2B) in NSCLC patients. To assess the influence of lymph-node metastasis and tumor size on prognosis of NSCLC patients, Kaplan-Meier survival curves were plotted and log rank analysis was performed. Lymph-node metastasis was significantly associated with shorter OS (*P* = 0.026; Fig 2C) and DFS (*P* = 0.002; Fig 2D) in NSCLC. Similar results were obtained for tumor size (OS, *P* < 0.001, Fig 2C and DFS, *P* < 0.001, Fig 2D).

**Fig 3:**
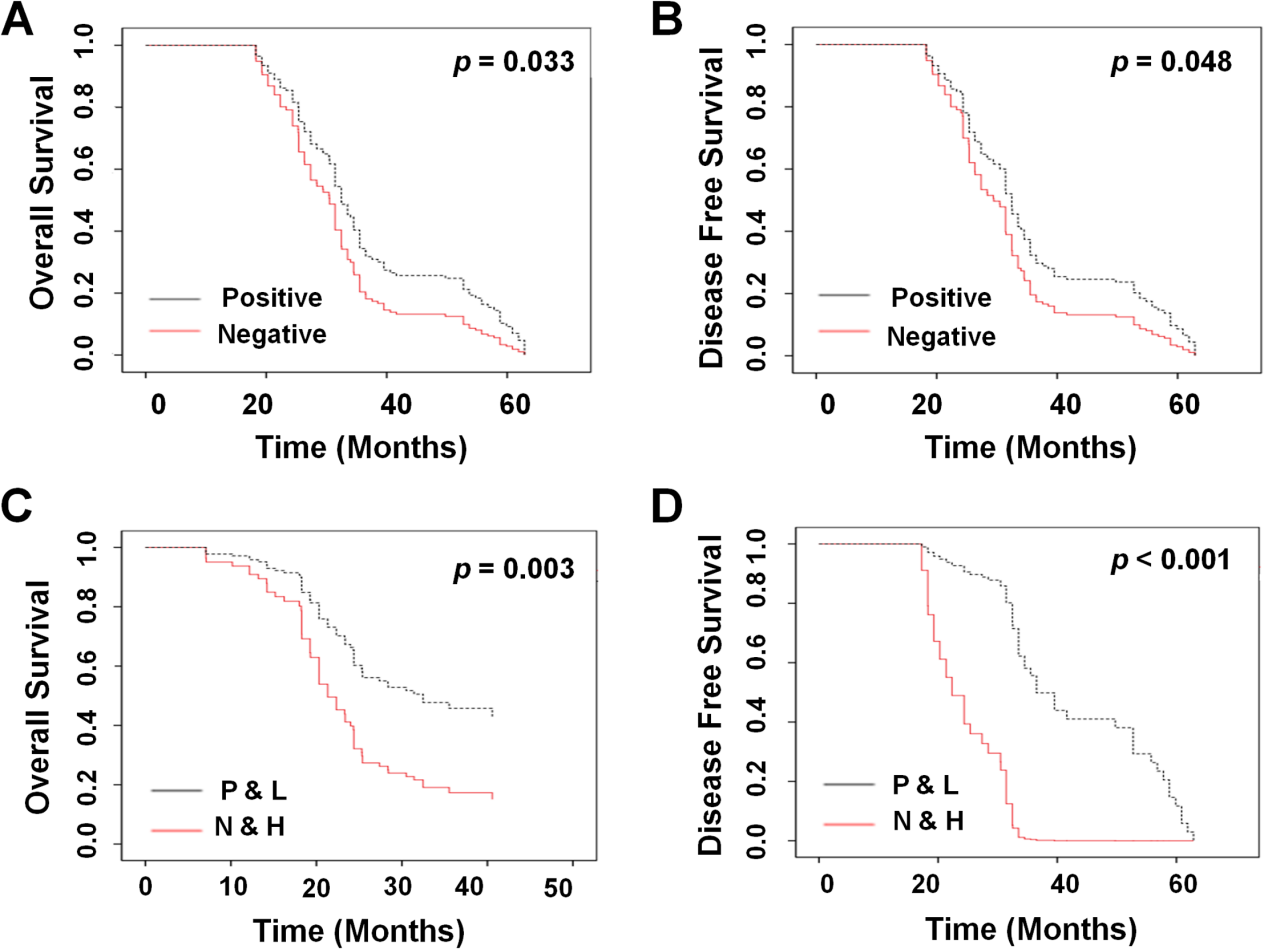
Survival analysis of chemotherapy and *TGFβR2* gene expression in NSCLC. Univariate analysis of OS (A) and DFS (B) with a Cox proportional hazards model in lung carcinoma based on chemotherapy, as determined by Cox regression estimates. Multivariate analysis of OS (C) and DFS (D) with a Cox proportional hazards model in lung carcinoma based on chemotherapy and *TGFβR2* expression, as determined by Cox regression estimates.

Univariate analysis with a Cox proportional hazards regression model revealed that invasion of lung membrane (*P* = 0.04), tumor size (*P* < 0.001), and lymph node metastasis (*P* = 0.02) were also positively correlated with poor prognosis (Table 3). More importantly, chemotherapy was found to significantly increase OS (*P* = 0.033, HR = 1.489 [1.032, 2.418], Fig 3A) and DFS time (*P* = 0.048, HR = 1.444 [1.004, 2.078], Fig 3B). In addition, low expression of *TGFβR2* in NSCLC tumors from patients treated with chemotherapy was a critical protective factor for OS (*P* = 0.003, HR = 2.24 [1. 32, 3.79], Fig 3C) and DFS (*P* < 0.001, HR = 9.40 [4.92, 17.98], Fig 3D). No correlations were observed with gender, age, vascular invasion, invasion of lung membrane, TNM stage, tumor differentiation, or smoking history. Taken together, these findings indicated that expression of *TGFβR2* might play a potential role in NSCLC progression and correlate with the outcome of NSCLC patients.

**Table 3:** Cox regression model analysis for prognosis based on various clinical characteristics in NSCLC patients.

Factor	HR	95% CI (univariate)	P value		TGFBR2 multivariate analysis			
				HR	95% CI (multivariate)	P value
Age	0.94	0.62–1.43	0.77			
Gender	0.68	0.42–1.08	0.10			
Smoking history	0.96	0.60–1.50	0.85			
Lymph-node metastasis	1.66	1.07–2.57	0.02	1.58	1.01–2.46	0.04
Tumor differentiation	0.9	0.59–1.37	0.62			
Histology	1.48	0.97–2.26	0.34			
TNM stage	1.34	0.83–2.17	0.22			
Invasion of lung membrane	1.73	1.03–2.87	0.04	1.72	1.03–2.88	0.03
Vascular invasion	3.97	0.55–28.93	0.55			
Diameter	3.73	2.32–5.79	<0.001	3.43	2.14–5.50	<0.001
TGFBR2 expression	1.81	1.51–2.86	0.01			

A multivariate Cox proportional hazards regression analysis was carried out to establish if expression of *TGFβR2* was a prognostic marker in NSCLC patients. The model initially included all of the parameters that were predictive of OS in the univariate analysis of the entire study group as presented in Table 3 (age, gender, smoking history, lymph-node metastasis, tumor differentiation, histology, vascular invasion and diameter, and invasion of lung membrane). A forward stepwise procedure was adopted to obtain the final model of significant predictors for OS consisting of the factors lymph-node metastasis, diameter, invasion of lung membrane, and expression of *TGFβR2*. According to multivariable Cox regression model analysis, high expression of *TGFβR2* was identified as a predictor of shorter OS in NSCLC patients.

### Chemotherapy associated with low expression of TGFβR2 highly improves OS and DFS of NSCLC patients

Chemotherapy serves as the primary treatment in the majority of NSCLC cases. The OS and DFS of patients were therefore analyzed based on treatment status. Chemotherapy was found to significantly prolong OS (33.5±1.05 vs. 24.37 ± 1.22, *P* = 0.025) and DFS (32.47 ± 0.84 vs. 24.37 ± 1.22, *P* = 0.037) of the patients in this cohort (Table 4). When the data was analyzed based on *TGFβR2* expression as well as treatment status, OS and DFS time was found to be significantly longer in treated NSCLC patients with low expression of *TGFβR2* as opposed to untreated patients with high expression (40.70 ± 2.07 vs. 24.23 ± 1.25, *P* = 0.002 and 36.53 ± 2.97 vs. 21.33 ± 1.34, *P* < 0.001) (Table 4).

**Table 4:** OS and DFS of NSCLC patients based on chemotherapy alone or chemotherapy and TGFβR2 expression.

			OS					DFS			
		Mean ± SD	95% CI	P value	Mean ± SD	95% CI	P value
Chemotherapy	Positive	33.50 ± 1.05	31.43–35.56	0.025	32.47 ± 0.84	30.83–34.11	0.037
	Negative	24.37 ± 1.22	21.97–26.76		24.37 ± 1.22	21.97–26.76	
Chemotherapy & Expression	P & L *	40.70 ± 2.70	35.40–45.99	0.002	36.53 ± 2.97	30.70–42.35	<0.001
	N & H ^#^	24.23 ± 1.25	21.78–26.69		21.33 ± 1.34	18.69–23.96	

* P & L, Chemotherapy and TGFβR2 low expression

^#^ N & H, no Chemotherapy and TGFβR2 high expression.

The results demonstrated that *TGFβR2* was highly expressed in patients with a poor prognosis even when treated with chemotherapy. Univariate and multivariate survival analysis was conducted with Kaplan-Meier estimates to further determine whether chemotherapy and/or *TGFβR2* expression was associated with OS and DFS. The results of univariate analysis demonstrated that prolonged OS and DFS was associated with treatment (Fig 3A (HR = 1.489 [1.032, 2.418], *P* = 0.033) and Fig 3B (HR = 1.444 [1.004, 2.078], *P* = 0.048), respectively). When patient data were further stratified based on *TGFβR2* expression as well as treatment status, multivariate Cox proportional hazards regression analysis revealed an increased OS (HR = 2.24 [1.32, 3.79], *P* = 0.003, Fig 3C) and DFS (HR = 9.40 [4.92, 17.98], *P*< 0.001, Fig 3D) in treated patients with low *TGFβR2* expressing tumors.

## Discussion

The molecular components of the TGF-β family have been the focus molecular studies elucidating somatic mutations in many human cancers. Here, NSCLC biopsies and adjacent non-cancerous tissues were examined for expression of *TGFβR2*. The overall survival in patients with high expression (> 1.99 fold) of *TGFβR2* was decreased relative to patients with low expression of *TGFβR2*. In addition, clinical parameters such as lymph node metastasis and tumor size were associated with a worse prognosis in this cohort. Finally, an association between *TGFβR2* expression and chemotherapy emerged from our analysis, suggesting a possible role for this biomarker in the response to chemotherapy.

Resistance to chemotherapy is a major challenge in the treatment of cancer [24]. Cancer recurrence is an issue in patients not only under treatment with traditional chemotherapies but also with more contemporary molecular targeted therapies. Inhibition of the TGF-β signaling pathway with small-molecule compounds, however, has proven to be one way to reverse resistance in NSCLC to targeted therapy [25]. Several trials revealed that anti-*TGFβR2* antibody as a therapeutic inhibitor in combination with chemotherapy blocked the binding of *TGFβ1*, 2, and 3. Inhibition of TGF-β disrupted activation of Smad2, which has been shown to control metastasis, tumor growth, and the invasion of cancer cells [26]. Treatment with specific antibodies in other studies also inhibited primary tumor growth and metastasis [27,28]. Treatment with the cytotoxic agent cyclophosphamide in combination with inhibition of the TGF-β pathway was particularly effective in attenuating primary tumor growth and metastasis [26]. Therefore, a therapeutic approach that inhibits *TGF-β* signal transduction might turn out to be specifically effective for NSCLC patients with detectable expression of *TGFβR2*. In fact, blocking TGF-β signal with soluble TGF-β receptor type II protein inhibited TGF-β binding to endogenous *TGF-β* receptors and reduced tumor cell motility, intravasation, and distant metastasis in a mouse model [29].

Clinicians have examined the utility of chemotherapy and radiation therapy in both preoperative and postoperative contexts in order to evaluate overall survival and disease free survival for patients with resectable NSCLC [30]. However, with an increasingly detailed molecular understanding of cancer development, individualized treatment strategies have become the focus for improvement of cancer therapy. Molecular biomarkers thus have begun to play a prominent role in the clinical management of cancer patients, as they indicate the probability of a response to chemotherapeutic intervention in individual patients. Biomarkers currently relevant for targeted therapy in NSCLC are EGFR, HER2, and KRAS mutational status, ALK gene rearrangement, and c-Met protein expression [31]. These biomarkers have stimulated the co-development of new drugs with companion diagnostics [32].

Significant effort to identify a molecular signature that would predict response to cancer adjuvant therapy is ongoing in order to conduct and improve the current standard of clinical care [33–35]. However, the real potential for biomarkers lies in their possible development as a molecular targeted therapy in the treatment of specific subsets of NSCLC patients [36]. Many crucial signaling pathways are involved in the development of NSCLC, and chemotherapy sensitivity or resistance to various antibody or inhibitor agents that target these pathways can be specifically administered based on the appearance of specific biomarkers in individual patients [26,37,38].

In summary, our data demonstrated that with chemotherapeutic treatment the median OS and DFS of patients were increased. In addition, patients stratified by chemotherapy together with low expression of *TGFβR2* exhibited the longest OS and DFS in this cohort. In contrast, untreated patients with high expression of *TGFβR2* exhibited the shortest OS and DFS. The analyses thus demonstrated that in the case of low *TGFβR2* expression, chemotherapy greatly improves overall survival and disease free survival in NSCLC patients. Therefore, *TGFβR2* is a potential tumor biomarker for chemosensitivity in NSCLC. However, further study with larger cohorts is necessary to confirm these findings.

## Conclusion

*TGFβR2* expression in NSCLC tissues was significantly higher than in non-neoplastic tissues. Analyses revealed that high expression of *TGFβR2* (> 1.99) was a critical risk factor for reduced OS and DFS in NSCLC patients. Therefore, our findings indicate that *TGFβR2* transcript levels may have an important role in NSCLC progression and could develop as a promising prognostic biomarker for patients with NSCLC chemotherapy.
